# Health-related quality of life and fatigue in patients with chronic hepatitis C with therapy with direct-acting antivirals agents interferon-free

**DOI:** 10.1371/journal.pone.0237005

**Published:** 2020-08-19

**Authors:** Raíssa Neves Fagundes, Lincoln Eduardo Villela Vieira de Castro Ferreira, Fábio Heleno de Lima Pace

**Affiliations:** Department of Gastroenterology, University Federal of Juiz de Fora, Juiz de Fora, MG, Brasil; Centers for Disease Control and Prevention, UNITED STATES

## Abstract

**Introduction:**

Interferon (IFN)-free regimens for the treatment of chronic hepatitis C have shown high rates of sustained virological response (SVR) and improved patient-reported outcomes (PROs). The aim of this study was to evaluate the health-related quality of life (HRQoL) and fatigue of patients with chronic hepatitis C (HCV) treated with IFN-free direct-acting antiviral (DAA) agents that achieved SVR following treatment and identify the predictive factors related to HRQoL.

**Methods:**

Prospective cohort study that included patients with HCV treated with DAA who obtained an SVR. The patients answered three self-reported questionnaires (PROs): Short Form 36 (SF-36), the Chronic Liver Diseases Questionnaire (CLDQ), and the Functional Assessment of Chronic Illness Therapy-Fatigue (FACIT-F) questionnaire at baseline, weeks 6 and 12 of treatment, and at 12 weeks after therapy. Patients were treated with DAA with or without ribavirin (RBV). The PRO scores were compared using analysis of variance (ANOVA). A comparison of PROs and serum hemoglobin levels was performed between the group that used ribavirin and the one that did not use ribavirin using the t student test. Predictive factors were calculated using a multiple linear regression model.

**Results:**

Among the 113 patients selected, 105 presented an SVR and were included in the study, in which, 54% men, 80% genotype 1, 44% cirrhosis and 46% with RBV. At 12 weeks after the end of treatment, there was a significant improvement in the scores of the patient self-reports (PROs) when compared with baseline for the CLDQ (+10.52%, p<0.001), SF-36-Physical Summary (+19%, p<0.001), and FACIT (+17.34%, p<0.001). Patients who used RBV had worse PROs and serum hemoglobin levels compared to the group that did not use RBV (p<0,05). As predictors of worsening of the PROs we had the presence of diabetes mellitus, liver cirrhosis and HIV co-infected.

**Conclusion:**

Patients treated with IFN free regimens presents significant improvement in PROs. The presence of diabetes mellitus, cirrhosis, and HIV co-infected has a negative effect on HRQoL before, during and after treatment of hepatitis C. The addition of ribavirin to the antiviral regimens used compromises the HRQoL indexes during antiviral therapy.

## Introduction

Chronic hepatitis C virus (HCV) infection affects 70 million people worldwide [1]. Chronic diseases have a negative impact on patients’ sense of psychological and physical well-being. Chronic hepatitis C can significantly compromise health-related quality of life (HRQoL) indices that represent the effect of the disease and treatment on patients’ physical and emotional roles [2–4]. Significant fatigue and a consequent decline in productivity and labour capacity are also observed more frequently in this population [5].

Interferon (IFN)-based treatments have been shown to be associated with impairment of quality of life indices during therapy [6]. The introduction of direct-acting antiviral (DAA) drugs has revolutionized chronic hepatitis C treatment, as they are drugs with few adverse events and a high rate of sustained virological response (SVR) [7–9]. A study published by Younossi [10] evaluated the HRQoL of 1005 patients with chronic hepatitis C, in different stages of liver fibrosis treated with sofosbuvir (SOF) and ledipasvir and found a significant improvement in HRQoL after obtaining an SVR [1].

In Brazil, in 2015, the second-generation DAAs SOF, simeprevir (SIM), and daclatasvir (DCV) were incorporated into the Clinical Protocol and Therapeutic Guidelines (CPTG) for Hepatitis C and Coinfections of the Ministry of Health of Brazil [11]. However, studies on the impact of these new drugs on HRQoL indices in real life patients are scarce. Therefore, the aim of this study is to analyse whether IFN-free therapeutic regimens (SOF, SIM and DCV) with or without ribavirin (RBV) impact HRQoL.

## Methods

This was a cohort, observational study with prospective inclusion of data; patients with chronic hepatitis C being followed through the Division of Hepatology of the Gastroenterology Service of the University Hospital of the Federal University of Juiz de Fora (Hospital Universitário da Universidade Federal de Juiz de Fora—HU-UFJF) during the period from April 2016 to February 2018, aged between 18 and 75 years, who received IFN-free antiviral therapy according to the recommendations of the clinical protocol and therapeutic guidelines (CPTG) for Viral Hepatitis C and Coinfections of the Brazilian Ministry of Health of 2017 and who obtained an SVR were included [11]. Patients diagnosed with hepatocellular carcinoma and decompensated cirrhosis were excluded.

According to the CPTG recommendations, patients with genotype 1 were treated with SOF 400 mg/day and DCV 60 mg/day or SOF and SIM 150 mg/day (oral) with or without RBV for 12 weeks. Patients with genotype 3 received SOF 400 mg/day and DCV 60 mg/day with or without RBV for 12 weeks. A negative viral load 12 weeks after the end of therapy was considered an SVR.

According to the protocol, patients treated for 12 weeks were clinically evaluated at baseline (before the start of treatment), at weeks 6 and 12 of treatment (treatment end), and at 12 weeks after the end of treatment. Hemoglobin, alanine aminotransferase (ALT), aspartate aminotransferase (AST) were assessed at same intervals. Patients were considered cirrhotic by clinical criteria (signs of liver decompensation), laboratory and / or imageological tests such as the AST to platelet ratio index (APRI), FIB-4 or fibroscan or by liver biopsy or by hepatic elastography when available. The degree of liver dysfunction was classified according to the Child-Pugh and MELD scores. The study protocol was approved by the Research Ethics Committee of HU-UFJF, Brazil, CAAE: 55880516.0.0000.5133. Patients were included in the research after reading and signing the informed consent form.

### Evaluation of PROs (patient-reported outcomes)

To evaluate the PROs, the patients were interviewed at the following four moments by the same researcher before the clinical assessment by the attending physician: baseline, weeks 6 and 12 of treatment, and 12 weeks after the end of treatment. This is a double-blind study, in which the patient and the researcher at the time of the interview were unaware of the results of the patient's laboratory and clinical exams. All self-assessment instruments were previously validated in Brazil.

The Short Form 36 (SF-36) and the Chronic Liver Diseases Questionnaire (CLDQ) were used to measure HRQoL. The SF-36 consists of 11 questions and 36 items comprising eight domains: physical functioning (PF), role physical (RP), bodily pain (BP), general health (GH), vitality (VT), social functioning (SF), role emotional (RE) and mental health (MH). To analyse the results, the eight domains were grouped into two major components: Physical Component Summary score (PCS) and Mental Component Summary (MCS). A score ranging from 0 to 100 was provided for each domain, with 0 being the worst score and 100 being the best score [12]. This questionnaire was previously validated in Brazil by Ciconelli et al, in 1999 [12].

The CLDQ is a specific questionnaire for liver disease and consists of 29 items grouped into six domains: abdominal symptoms (AS), fatigue (FA), systemic symptoms (SS), activity/energy (ACTI), emotional (EM) and worry. The scores calculated for each domain range vary from 1 (worst) to 7 (best). Higher scores indicate a minimal frequency of symptoms and, consequently, better HRQoL. The total score is calculated as the mean value of the 29 items [13]. This questionnaire was previously validated in Brazil by Mucci et al, in 2010 [13].

Fatigue was assessed using the Functional Assessment of Chronic Illness Therapy-Fatigue (FACIT-F) questionnaire, validity in Brazil to assess fatigue in patients with cancer, arthritis and other diseases [14]. This questionnarie includes four independent HRQoL domains: physical (PWB), emotional (EWB), social (SWB), functional (FWB), and fatigue subscale (FS). The PWB, SWB, and FWB domains range from 0 to 28, the EWB domain ranges from 0 to 24, and the FS domain ranges from 0 to 52. The set of five domains results in the FACIT-F total score, which ranges from 0–160 [14]. The Portuguese version was used with authorization from the website http://www.facit.org.

### Statistical analysis

SPSS 21.0 software (SPSS, Chicago, IL) was used for statistical analyses. Nonparametric variables are expressed as the median, and those with a normal distribution are expressed as the mean±standard deviation (SD). To assess the impact of antiviral therapy on PROs, the scores measured were compared before treatment, at weeks 6 and 12 of treatment, and 12 weeks after the end of treatment using repeated measures ANOVA, in which p<0.05 was established as the level of significance. To evaluate the effect of treatment on PRO scores, the differences between the mean scores for the 6th week of treatment, 12th week of treatment, and 12 weeks after the end of treatment and the mean baseline scores were also calculated and were compared using Student t-tests.

To assess the impact of ribavirin (RBV) on PROs, patients were divided into two groups: patients treated with RBV and without RBV. All PROs were compared between the two groups before treatment, at weeks 6 and 12 of treatment, and 12 weeks after the end of treatment using t Student test, in which p<0.05 was established as the level of significance. The serum hemoglobin levels of the two groups were compared before treatment, at weeks 6 and 12 of treatment, and 12 weeks after the end of treatment using t Student test, in which p<0.05 was established as the level of significance.

A multiple linear regression model was used to identify factors independently associated with HRQoL. The following variables were included in the analysis: age, sex, previous exposure to treatment, RBV use, cirrhosis presence, HIV co-infection, diabetes and presence of hypertension. A positive β coefficient indicated an improvement and negative β coefficient indicated a decline in PROs; the level of significance was p<0.05.

## Results

A total of 113 patients were selected, of whom 105 (96%) had an SVR and were included in the study. The sample consisted of 57 (52,29%) men with the mean age of 58.69±9.88 years. SOF/DCV was the scheme used by 63 (60%) patients, and 42 (40%) received SOF/SIM with or without RBV. There was a predominance of genotype 1 (80%), 60% were untreated, and 44% were cirrhotic. The main clinical, demographic and laboratory characteristics are provided in Table 1.

**Table 1:** Clinical, socio-demographic and patient characteristics in pre-treatment.

Parameters	Absolute frequency	Relative frequency
**Gender (F/M)**	48 / 57	47.71% / 52.29%
**Age (years)**	58.69 ± 9.88	
**Genotype 1/2/3**	84 / 1 / 20	80% / 0.95% / 19.05%
**Not experienced**	64	60.95%
**HIV positive**	16	15.24%
**Cirrhosis**	46	43.81%
**DM2**	13	12.38%
**Hypertensive**	36	34.28%
**SOF/SIM**	31	29.52%
**SOF/DCV**	26	24.76%
**Use of ribavirin**	48	45.71%
**ALT (U/L)**	85.41 ± 65.27	
**AST (U/L)**	87.66 ± 57.98	
**Hemoglobin (mg/dL)**	14,01 ± 1,86	

Absolute frequency. Relative frequency. Mean ± standard deviation. N = 105. F, female; M, male; HIV, human immunodeficiency virus; DM2, type 2 diabetes mellitus; SOF, sofosbuvir; SIM, simeprevir; DCV, daclatasvir; ALT, alanine aminotransferase; AST, aspartate aminotransferase.

### HRQoL according to the SF-36 and CLDQ

**Fig 1:**
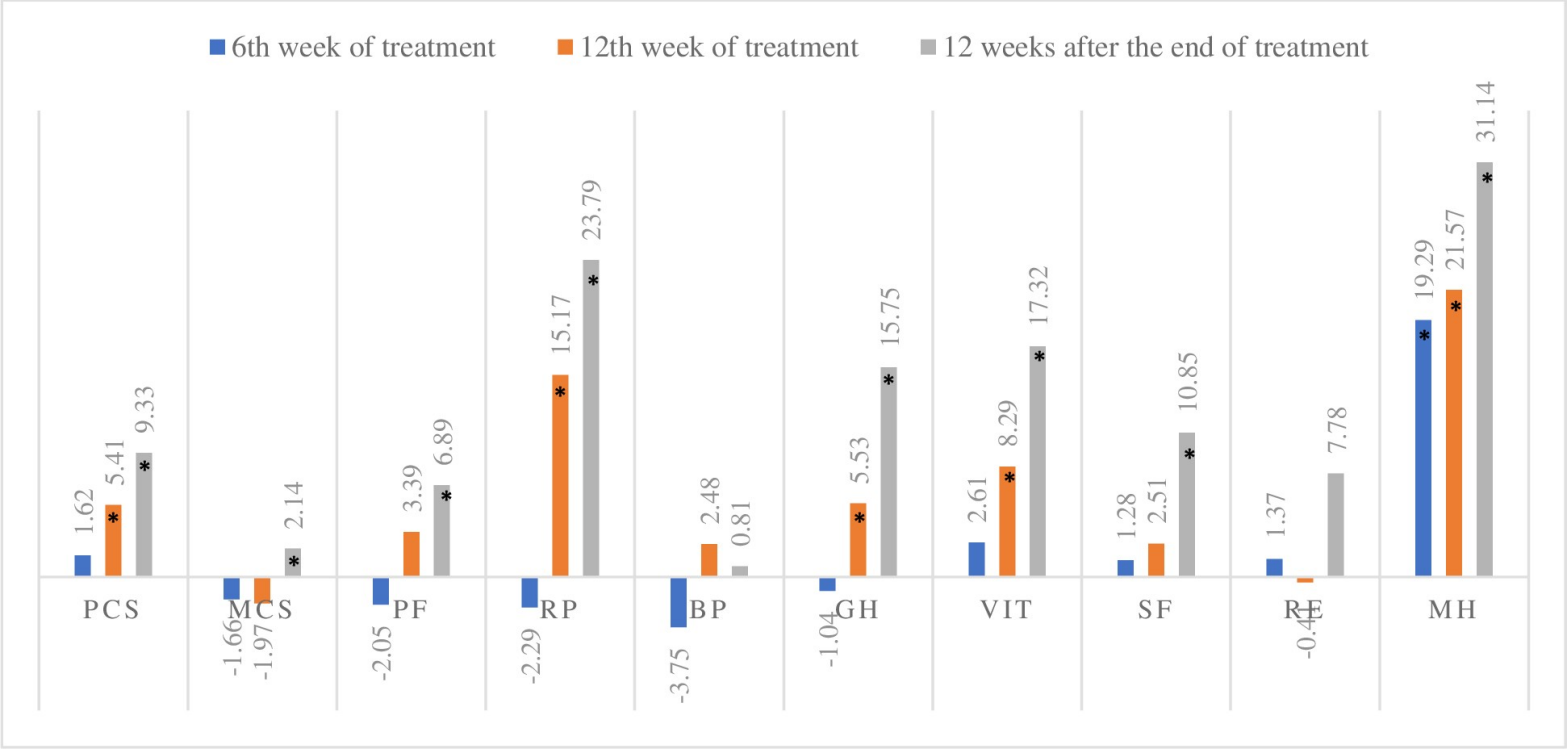
Difference of the mean score in relation to the SF-36 baseline. Analysis of Student t test to compare the means of week 6 of treatment, week 12 of treatment and 12 weeks after the end of treatment in relation to the baseline scores, * p <0.05. N = 105. PCS, Physical Component Summary; MSC, Mental Summary Component; PF, physical functioning; RP, role physical; BP, bodily pain; GH, general health; VIT, vitality; SF, social functioning; RE, role emotional; MH, mental health.

There was improvement in the HRQoL indices assessed by the SF-36 and CLDQ (Table 2). Regarding the generic SF-36 questionnaire, the PCS (p <0.001) and the MCS (p = 0.04) improved significantly. There was an improvement in PROs during treatment and 12 weeks after the end of treatment in 6 of the 8 domains: physical capacity (p <0.001), limitations due to physical aspects (p <0.001), general health status (p <0.001), vitality (<0.001), social aspects (p <0.001) and mental health (p <0.001) (Table 2). At 12 weeks after the end of treatment, PCS and MCS showed an increase of 19% (+9.33, p<0.001; r = 0.42) and 4% (+2.14, p = 0.04; r = 0.16), respectively, when compared to baseline. For the SF-36, an improvement in PROs was observed in seven of the eight domains (p<0.001). The domains role physical and vitality showed an increase of 33.19% and 25.97%, respectively, when comparing the scores at baseline with the scores 12 weeks after the end of treatment (Fig 1).

**Table 2:** HRQoL assessed by different instruments before, during, and after hepatitis C treatment.

Instrument/Domain	Baseline	Week 6	Week 12	12 weeks after treatment	*p* value
**SF-36**					
Physical functioning*	77.89	75.84	81.28	84.78	<0.001
**Role physical** *	71.68	68.76	86.85	95.43	<0.001
Bodily pain*	83.13	79.38	85.61	83.94	<0.001
General health*	60.23	59.19	65.54	75.95	<0.001
Vitality*	66.77	69.38	75.06	84.09	<0.001
Social functioning*	78.62	79.9	81.13	89.47	<0.001
**Role Emotional**	81.19	82.56	80.78	88.77	>0.05
Mental health*	48.05	67.34	69.62	79.19	<0.001
Physical Component Summary*	47.77	49.39	53.18	57.10	<0.001
Mental Component Summary*	48.27	46.61	46.31	50.41	= 0.04
**CLDQ**					
Abdominal symptoms*	6.17	6.12	6.47	6.48	= 0.02
Fatigue*	5.86	5.68	6.10	6.81	<0.001
Systemic symptoms*	5.88	5.87	6.29	6.55	<0.001
Activity*	6.24	6.15	6.45	6.84	<0.001
**Emotional function**	5.52	5.60	5.66	5.88	>0.05
Worry*	6.39	6.54	6.71	6.83	<0.001
Total–CLDQ*	5.99	6.03	6.34	6.63	<0.001
**FACIT-F**					
Total FACIT-F*	43.32	43.12	45.23	49.69	<0.001
Fatigue*	126.84	123.28	133.25	148.84	<0.001
Physical well-being*	24.04	22.32	26.21	26.29	= 0.023
Social well-being*	22.62	20.06	21.58	24.27	<0.001
Emotional well-being*	17.06	19.97	19.67	21.89	<0.001
Functional well-being*	19.55	18.59	21.51	22.24	<0.001

ANOVA test for evaluation between the 4 visits.

*p<0.05. N = 105. SF-36, short form– 36; CLDQ, chronic liver diseases questionnaire; FACIT-F, functional assessment of chronic illness therapy-fatigue.

**Fig 2:**
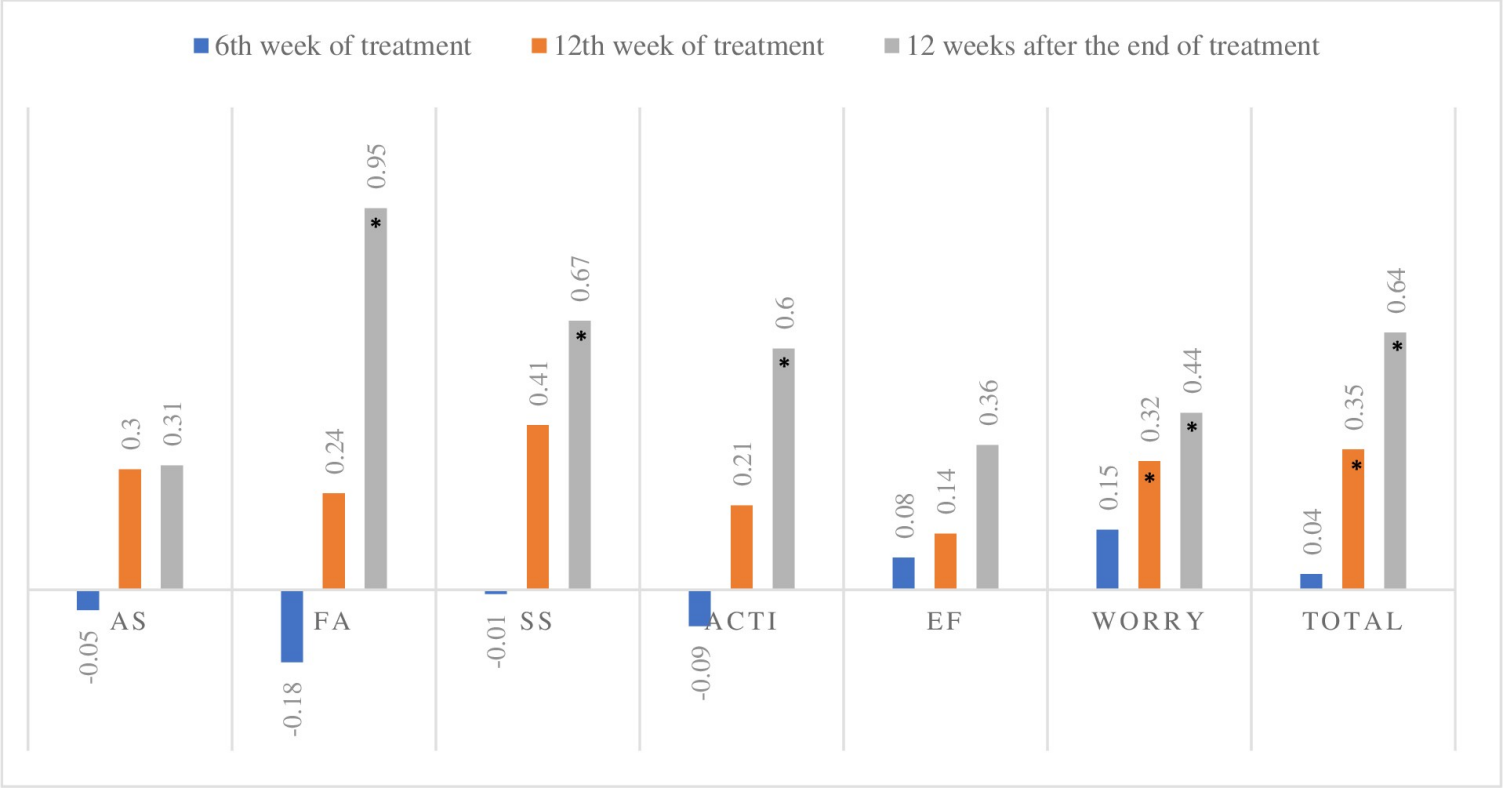
Difference of the mean score in relation to the CLDQ baseline. Analysis of Student t test to compare the means of week 6 of treatment, week 12 of treatment and 12 weeks after the end of treatment in relation to the baseline scores, * p <0.05. N = 105. AS, abdominal symptoms; FA, fatigue; SS, systemic symptoms; ACTI, activity; EF, emotional function.

According to the CLDQ, a significant increase in PROs was observed after the institution of therapy. The total domain (mean of the six domains) showed progressive improvement after the 12th week of treatment. The scores obtained 12 weeks after the end of the treatment were significantly better when compared to baseline, 6th and 12th weeks of treatment. Among the 6 domains evaluated, 5 showed a statistically significant increase: abdominal symptoms (p = 0.02), fatigue (p <0.001), systemic symptoms (p <0.001), activity (p <0.001) and concern (p <0.001). The emotional function showed no significant difference (p> 0.05) (Table 2). The total domain, which is the sum of all domains, showed an increase of 10.52% (+0.63, p <0.000, r = 0.43) at the 12th week after the end of treatment when compared to baseline (Fig 2). Among the CLDQ domains, four showed significant improvement 12 weeks after the end of treatment compared to baseline: fatigue (+0.95, p <0.001, r = 0.43), systemic symptoms (+0.67, p <0.001, r = 0.38), activity (+0.6, p <0.001, r = 0.53) and worry (+0.44, p <0.001, r = 0.36) (Fig 2).

### Fatigue according to the FACIT-F

**Fig 3:**
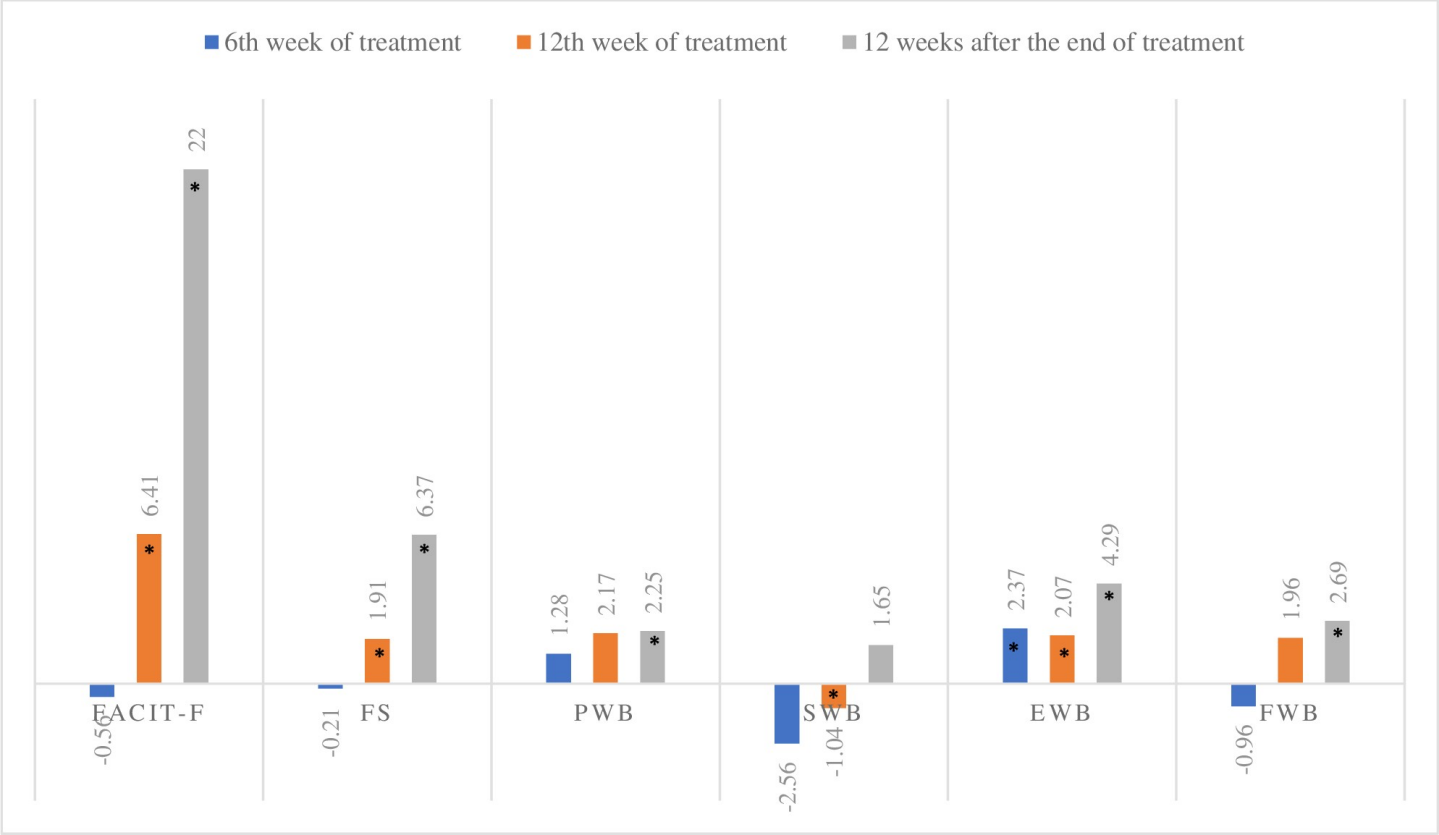
Difference of the mean score in relation to the FACIT-F baseline. Analysis of Student t test to compare the means of week 6 of treatment, week 12 of treatment and 12 weeks after the end of treatment in relation to the baseline scores, * p<0.05. N = 105. FACIT-F, functional assessment of chronic illness therapy-fatigue; FS, fatigue; PWB, physical well-being; SWB, social well-being; EWB, emotional well-being; FWB, functional well-being.

All domains that comprise the FACIT questionnaire showed a significant difference between the weeks evaluated: physical well-being (p = 0.023), emotional well-being (p <0.001), social well-being (p <0.001), functional well-being (p> 0.001) and fatigue (p <0.001) (Table 2). The fatigue symptoms evaluated using the FACIT-F showed a significant improvement at the end of treatment (+6.41, p <0.001 r = 0.34) and at 12 weeks after the end of treatment (+22.00, p <0.001, r = 0.47) when compared to the values obtained at baseline (Fig 3).

### Evaluation of the impact of ribavirin use on PROs

**Fig 4:**
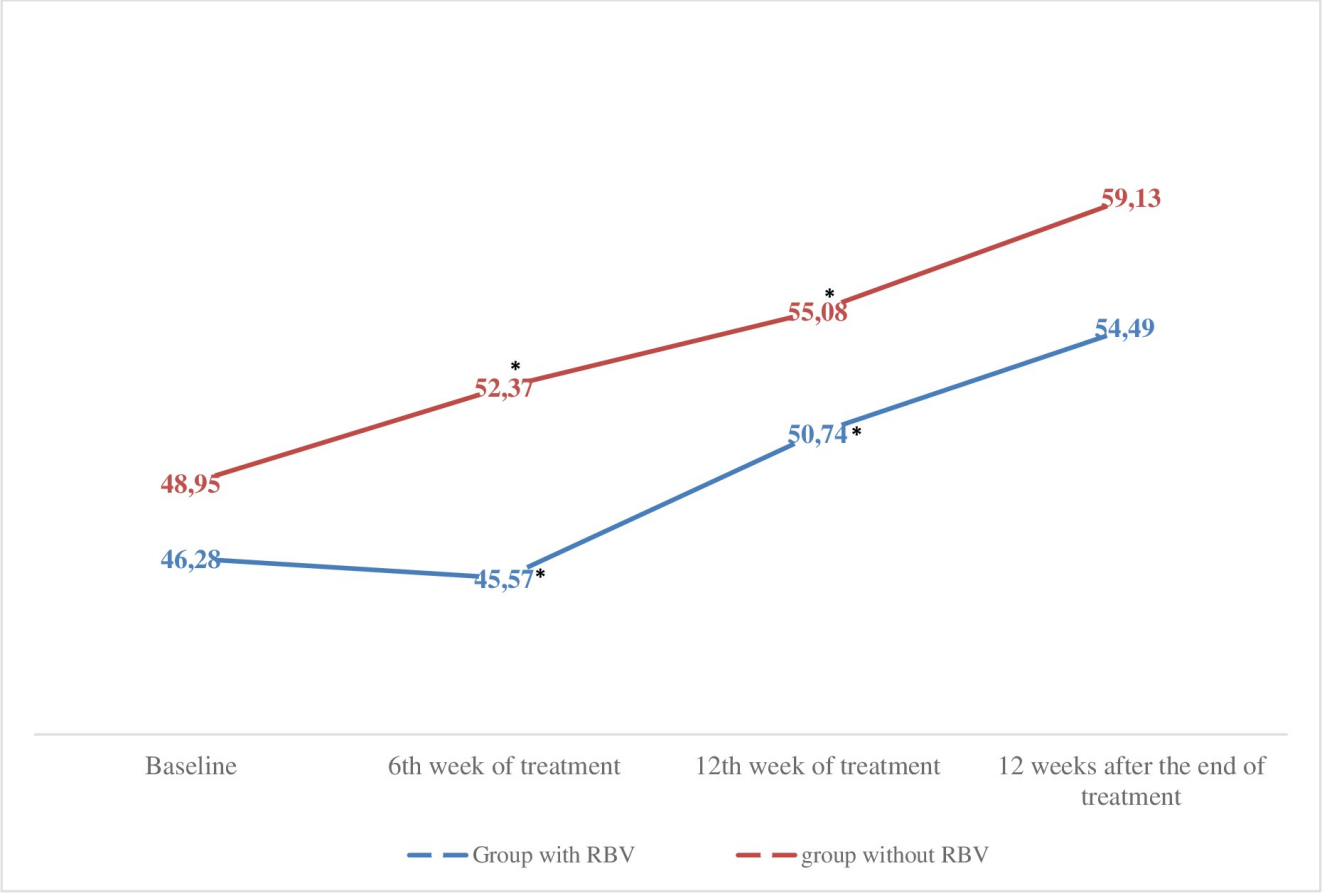
Physical Component Summary (SF-36) before, during and after the end of treatment in the group with and without ribavirin. Comparison of the averages analysed using the Student t test, * p <0.05. N = 105. RBV, ribavirin.

There was a significant difference in HRQoL and fatigue between the group that used ribavirin in the treatment and the group that did not use it (p <0.05). Regarding the SF-36 questionnaire before the beginning of treatment, there was no difference in the PROs of these two groups (p> 0.05), however, at week 6 of treatment, physical capacity (66.84 vs. 84.07, p = 0.002), pain (70.98 vs. 88.40, p = 0.000), vitality (63.30 vs. 76.44, p = 0.004), social aspects (73.069 vs. 85.24, p = 0.020) and the summarized physical component (45.57 vs. 52.37, p = 0.012) was significantly lower in the group that used ribavirin compared to the group that did not. At week 12 of treatment, physical capacity (75.087 vs. 85.50, p = 0.02), social aspects (74.24 vs. 86, 82, p = 0.012) and the summarized physical component (50.074 vs. 55.08, p = 0.039) was significantly lower in the group that used ribavirin than in the group that did not. There was no significant difference between groups after the end of treatment (p> 0.05) (Fig 4).

Regarding the analysis of the groups using the CLDQ questionnaire, it was possible to observe that there was no significant difference between the groups before the beginning of treatment (p> 0.05). At week 6 of treatment, the group that used ribavirin had lower scores than the group that did not use ribavirin in the following domains: systemic symptoms (5.61 vs. 6.10, p = 0.020) and activity (5.87 vs. 6, 47, p = 0.014). At week 12 of treatment, the domain: systemic symptoms (5.14 vs. 6.27, p = 0.021) obtained significantly lower scores in the group that used ribavirin than in the group that did not. There was no significant difference between groups after the end of treatment (p> 0.05).

**Fig 5:**
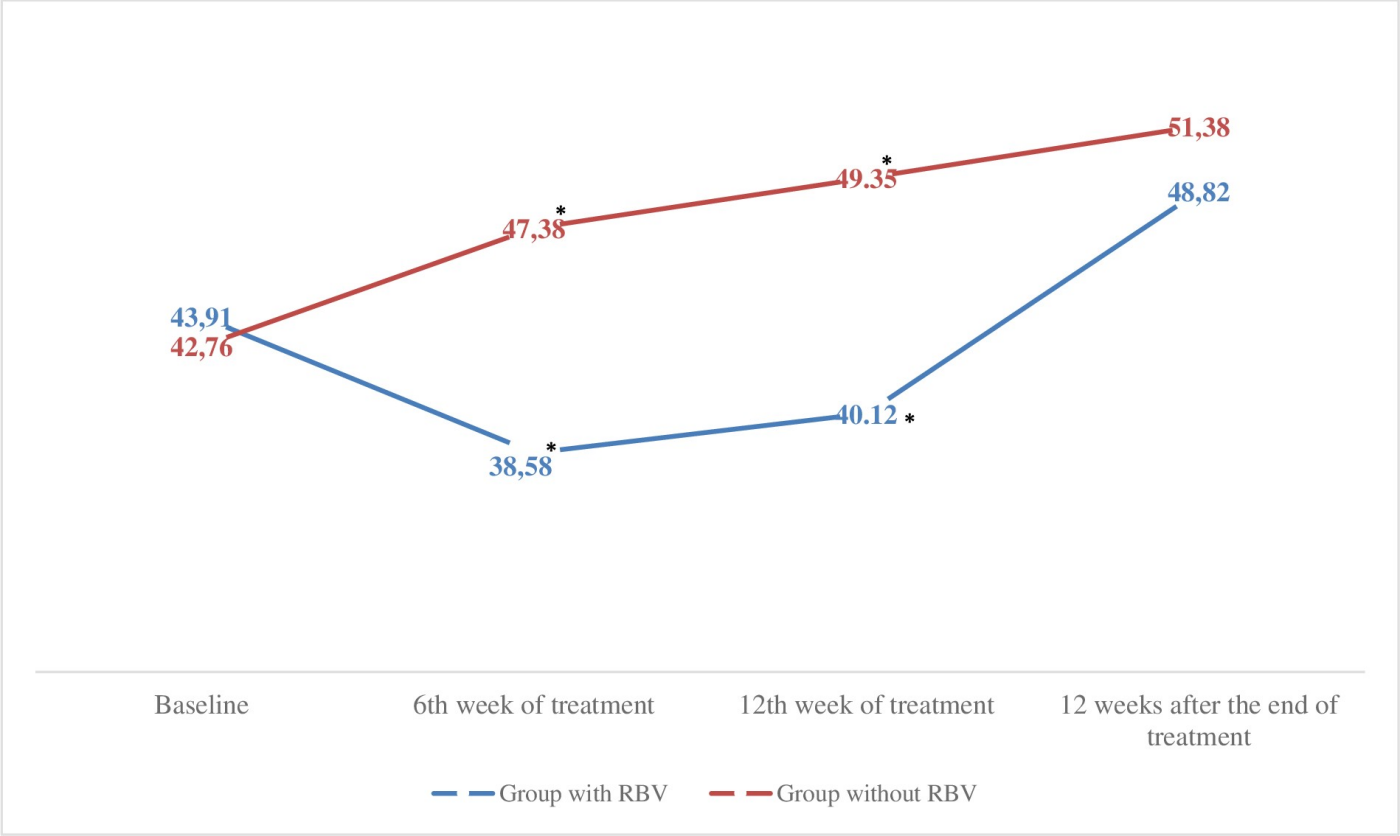
Fatigue (FACIT) before, during and after treatment in the group with and without ribavirin. Comparison of the averages analysed using the Student t test, * p <0.05. N = 105. RBV, ribavirin.

Regarding the FACIT questionnaire to assess fatigue, there was also no significant difference between groups before treatment (p> 0.05). At week 6 of treatment, the domains: functional well-being (16.23 vs. 20.42, p = 0.003), fatigue (38.58 vs. 47.38, p = 0.002) and the FACIT-F (114.08 vs. 132.05, p = 0.001) were significantly lower in the ribavirin group than in the ribavirin group. At week 12 of treatment, the fatigue domain (40.12 vs. 49.35, p = 0.015) was lower in the ribavirin group than in the ribavirin group. There was no significant difference in the FACIT domains after the end of treatment (p> 0.05) (Fig 5).

**Fig 6:**
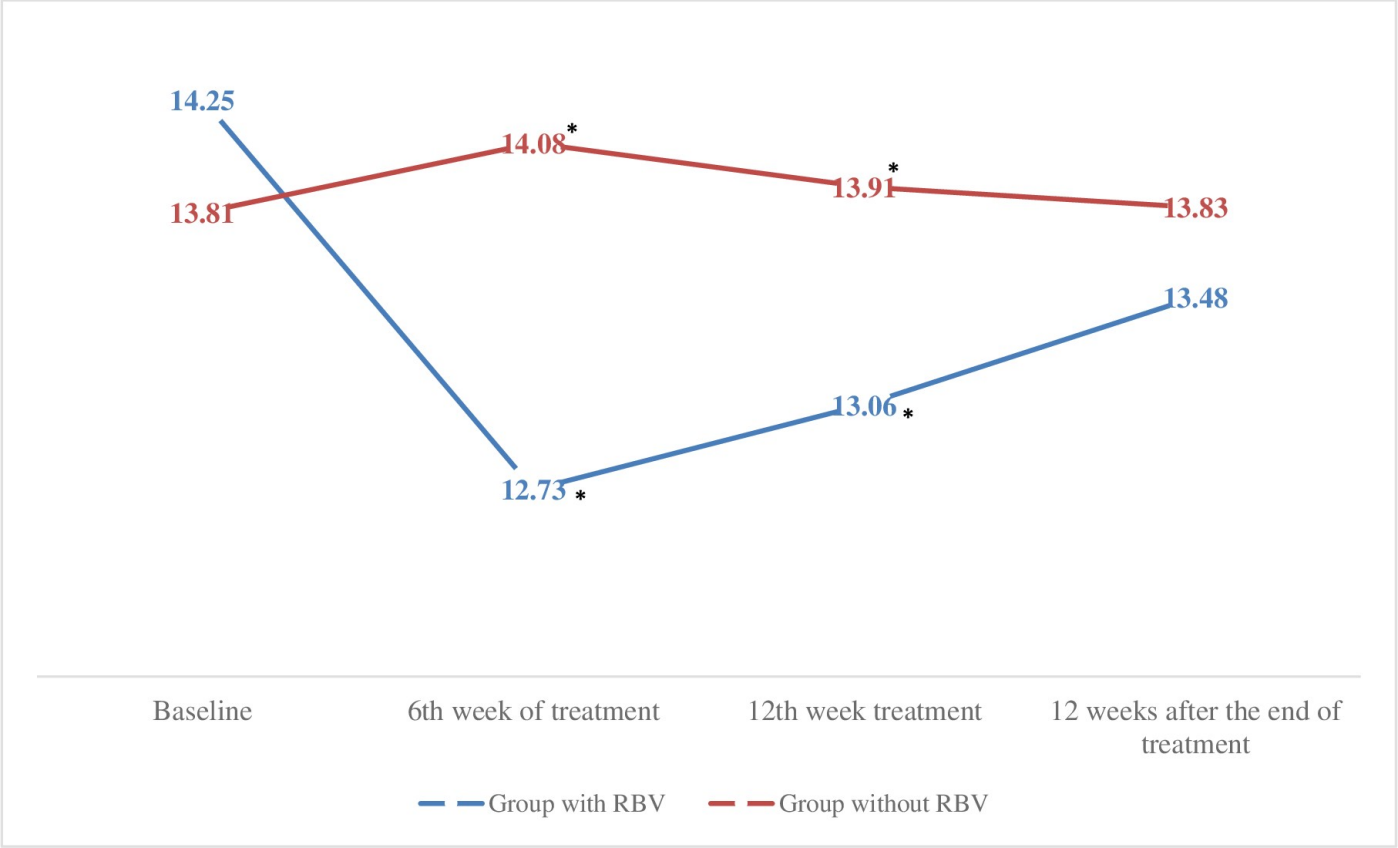
Evaluation of plasma hemoglobin levels before, during and after treatment in the ribavirin group and in the ribavirin group. Comparison of the averages analysed using the Student t test, * p <0.05. N = 105. RBV, ribavirin.

Patients who underwent therapy with AADs associated with RBV had lower plasma hemoglobin levels at week 6 (12.73 ± 2.21 vs. 14.08 ± 2.05, p = 0.001) and 12 (13, 06 ± 5.03 vs. 13.91 ± 2.11, p = 0.001) of treatment when comparing patients without association with RBV in treatment. There was no significant difference in plasma hemoglobin levels between the two groups before treatment (p> 0.05) and after the end of treatment (p> 0.05) (Fig 6).

### Predictors of HRQoL

Multiple linear regression demonstrated that the presence of diabetes mellitus was a predictor of worsening HRQoL in relation to physical and emotional symptoms before (p <0.05), during (p <0.05) and after the end of treatment p <0.05). The presence of cirrhosis was also a predictor of worsening HRQoL in relation to physical and emotional symptoms before (p <0.05), during (p <0.05) and after the end of treatment (p <0.05).

HIV positive was also a predictor of worsening HRQoL before (p <0.05), during (p <0.05) and after (p <0.05), in physical, emotional, and social symptoms.

The use of ribavirin associated with treatment with AADs was a predictor of worsening HRQoL during treatment (p <0.05).

## Discussion

In this study, we assessed the impact of IFN-free therapy on PROs in Brazilian patients with chronic hepatitis C who obtained an SVR after therapy with the DAAs SOF, DCV, and SIM. There was a significant improvement in HRQoL according to the SF-36 and CLDQ scores and in fatigue according to the FACIT-F at 12 weeks after the end of treatment compared to baseline. These findings are consistent with those reported by previously published studies that analysed HRQoL in patients with chronic hepatitis C treated with IFN-free regimens [1, 3, 7–9, 15–19].

The assessment of HRQoL was carried out using the generic questionnaire SF-36 and a specific questionnaire for patients with chronic liver disease, the CLDQ. Most domains assessed by SF-36 showed significant improvement 12 weeks after the end of treatment in relation to baseline scores, except for pain and limitations due to emotional aspects. In many studies, in patients with chronic hepatitis C, vitality is the most comprehensive measure of well-being [10]. In the present study, vitality showed a progressive increase in its scores after the 6th week of treatment. Although the HCV viral load was not made in the fourth week of therapy it is possible that in the sixth week there is no longer a viral replication of the HCV given the potency of the new therapeutic regimens. Regarding the CLDQ questionnaire, 4 of its 6 domains improved, 12 weeks after the end of treatment, except for emotional function and abdominal symptoms.

Fatigue is a common complaint in HCV patients and can be considered a debilitating symptom with a negative impact on quality of life [10, 20, 21]. In our study, fatigue symptoms, assessed by the FACIT-F questionnaire, increased by 22 points 12 weeks after the end of treatment compared to baseline, indicating an improvement in fatigue symptoms. In addition, the fatigue symptom, which was also analyzed using the CLDQ questionnaire, had a significant improvement after the end of treatment. According to Younossi et al., patients with chronic hepatitis C treated with sofosbuvir and ledipasvir showed significant improvement in fatigue after the end of treatment, according to the FACIT questionnaire. (+ 6.0% in CLDQ-HCV activity / energy, + 5.0% in FACIT-F fatigue score, + 6.8% in SF-36 physical component; all P <0.001) [17].

When assessing HRQoL, IFN-free regimens are clearly superior when compared to regimens containing IFNs. Fagundes et al. demonstrated that HRQoL indices are significantly compromised during antiviral therapy with pegylated IFN and the first-generation protease inhibitors telaprevir and boceprevir but that there was a significant improvement of HRQoL after SVR [6]. Regarding HRQoL, therapy with DAAs is clearly superior to therapy based on IFN [15, 22, 23]. Younossi et al, compared treatment with SOF associated or not with IFN. In this study, a decrease of -23.6% in the PROs was observed for the group with the IFN, while there was an improvement of up to + 11% in the free IFN regime [24]. Possibly, the high antiviral potency of IFN-free regimens leads to faster suppression of HCV replication and the absence, in most treated patients, of relevant adverse events justifies such findings.

In this study, it was possible to demonstrate that the recovery of HRQoL values occurs rapidly after the beginning of antiviral therapy and that in the twelfth week of treatment, the scores are significantly higher than those obtained at baseline. Although there was a drop in scores in the sixth week of treatment compared to baseline, this difference was not statistically significant (p> 0.05). It is possible that in the sixth week of treatment there will still be viral replication, so there is no significant improvement (p <0.05) when compared to the baseline. Gambalto et al, evaluated viral kinetics in 74 patients with hepatitis C during treatment with IFN-free DAAs, and observed that most patients obtained a viral load below 15UI / mL after 8 weeks of treatment [25].

The addition of RBV to antiviral regimens is still recommended for patients with predictive therapeutic failure factors, such as those who have not responded to previous treatments and those with decompensated cirrhosis [26, 27]. The most common adverse event related to the use of RBV is hemolytic anaemia [20]. It is questionable whether the combination of RBV with DAAs negatively impact HRQoL indices. In this study, patients treated with RBV had lower PROs when compared to those treated with RBV, in addition, these patients had lower serum hemoglobin levels during treatment (week 6 and week 12 of treatment) than the group that did not use RBV. Younossi et al. observed a decrease of 7.0% in the PROs of patients treated with SOF and RBV, while there was an increase of 11.6% in the group treated with SOF and ledipasvir (p<0.001) [17].

Among the predictors of worsening PRO in our study, diabetes mellitus, the presence of liver cirrhosis and HIV co-infection were negative predictors of HRQoL and fatigue before, during and after the end of treatment. Thuluvath et al., demonstrated that the presence of associated diseases in patients with chronic hepatitis C undergoing antiviral therapy significantly compromises the recovery of HRQoL indexes [16]. In these patients, it is possible that HCV eradication does not have sufficient power to recompose HRQOL indices that will continue to be compromised by the associated disease [16]. In addition, the use of therapeutic regimens containing RBV was independently associated with worse PROs. This again provides support for the superiority of RBV-free regimens.

The improvement observed in the well-being of cured patients, reinforces the idea that chronic hepatitis C is not a disease that exclusively affects the liver, but has a clearly systemic component capable of harming HRQoL [27].

The latest Brazilian guideline for the treatment of chronic hepatitis C, updated in 2019, includes new AADs such as sofosbuvir / ledipasvir, sofosbuvir / velpatasvir and glecaprevir / pibrentasvir [28]. The present study was developed from 2016 to 2018, and therefore, patients received the therapies contemplated in the current PCTD. As they are drugs of similar classes, it is possible that the findings regarding HRQoL are similar.

In our study, we had only 8 patients who did not obtain SVR, which limited the assessment of HRQoL among patients who obtained SVR and those who did not. This assessment is important to verify the effect of SVR on these patients' HRQoL. In addition, the assessment of the adverse events reported by the patients was not carried out, since this new treatment regime is known to have few adverse events.

In our study, we demonstrated the improvement of PROs in patients with real-life hepatitis C treated with IFN-free therapeutic regimens. HRQoL and fatigue had a progressive improvement during treatment and after SVR, showing that the eradication of the virus helps in better PRO scores. The presence of comorbidities in patients with hepatitis C, such as diabetes mellitus, is a negative predictor in PROs. Another negative prediction in the PROs was the presence of ribavirin in the therapeutic regimens. Such findings are consistent with those reported by studies previously published in other populations [1, 3, 7–9, 15–19, 22, 23].

## Conclusion

In summary, we demonstrated that IFN-free antiviral therapy with SOF, SIM and DCV significantly improved HRQoL in patients with chronic hepatitis C from week 6 of treatment until 12 weeks after the end of treatment. The presence of diabetes mellitus, cirrhosis, and HIV co-infected has a negative effect on HRQoL before, during and after treatment of hepatitis C. The addition of ribavirin to the antiviral regimens used compromises the HRQoL indexes during antiviral therapy.
